# *Coprinus comatus* Cap Inhibits Adipocyte Differentiation via Regulation of PPARγ and Akt Signaling Pathway

**DOI:** 10.1371/journal.pone.0105809

**Published:** 2014-09-02

**Authors:** Hyoung Joon Park, Jisoo Yun, Sun-Hee Jang, Suk Nam Kang, Beong-Sam Jeon, Yeoung-Gyu Ko, Hong-Duck Kim, Chung-Kil Won, Gon-Sup Kim, Jae-Hyeon Cho

**Affiliations:** 1 Institute of Life Science, College of Veterinary Medicine, Gyeongsang National University, Jinju, Korea; 2 Department of Molecular Science and Technology, Ajou University, Suwon, Korea; 3 Department of Animal Science & Biotechnology, Gyeongnam National University of Science and Technology, Jinju, Korea; 4 Animal Genetic Resources Station, National Institute of Animal Science, RDA, Namwon, Korea; 5 Department of Environmental Health Science, New York Medical College, Valhalla, New York, United States of America; Johns Hopkins University School of Medicine, United States of America

## Abstract

This study assessed the effects of *Coprinus comatus* cap (CCC) on adipogenesis in 3T3-L1 adipocytes and the effects of CCC on the development of diet-induced obesity in rats. Here, we showed that the CCC has an inhibitory effect on the adipocyte differentiation of 3T3-L1 cells, resulting in a significant decrease in lipid accumulation through the downregulation of several adipocyte specific-transcription factors, including CCAAT/enhancer binding protein β, C/EBPδ, and peroxisome proliferator-activated receptor gamma (PPARγ). Moreover, treatment with CCC during adipocyte differentiation induced a significant down-regulation of PPARγ and adipogenic target genes, including adipocyte protein 2, lipoprotein lipase, and adiponectin. Interestingly, the CCC treatment of the 3T3-L1 adipocytes suppressed the insulin-stimulated Akt and GSK3β phosphorylation, and these effects were stronger in the presence of an inhibitor of Akt phosphorylation, LY294002, suggesting that CCC inhibited adipocyte differentiation through the down-regulation of Akt signaling. In the animal study, CCC administration significantly reduced the body weight and adipose tissue weight of rats fed a high fat diet (HFD) and attenuated lipid accumulation in the adipose tissues of the HFD-induced obese rats. The size of the adipocyte in the epididymal fat of the CCC fed rats was significantly smaller than in the HFD rats. CCC treatment significantly reduced the total cholesterol and triglyceride levels in the serum of HFD rats. These results strongly indicated that the CCC-mediated decrease in body weight was due to a reduction in adipose tissue mass. The expression level of PPARγ and phospho-Akt was significantly lower in the CCC-treated HFD rats than that in the HFD obesity rats. These results suggested that CCC inhibited adipocyte differentiation by the down-regulation of major transcription factor involved in the adipogenesis pathway including PPARγ through the regulation of the Akt pathway in 3T3-L1 cells and HFD adipose tissue.

## Introduction

Obesity is associated with various diseases, particularly cardiovascular disease, type 2 diabetes, and metabolic diseases, including metabolic syndrome [1]. Obesity is a medical condition in which excess body fat has accumulated to such a high extent that it may cause adverse effects on health, leading to a reduced life expectancy and/or increased health problems. Thus, adipogenesis is closely related to the etiologies of obesity and obesity-related metabolic disorders [2]. Cellular and molecular studies concerning obesity have shown that increased numbers of adipocytes, which are associated with alterations in size, are triggered by genetic or dietary factors [3]. Increases in either adipocyte cell number or the size of the individual adipocytes, due to increased lipid accumulation and adipocyte differentiation, result in increased lipid deposition.

Adipocyte differentiation involves an elaborate network of transcription factors that regulate the expression of numerous genes responsible for the phenotypes of mature adipocytes [4]. The differentiation of preadipocytes into adipocytes is accompanied by a dramatic increase in the expression of CCAAT/enhancer binding protein β (C/EBPβ) and C/EBPδ, which transcriptionally activate the C/EBPα and peroxisome proliferator-activated receptor gamma (PPARγ) genes by binding to the C/EBP regulatory elements [5], [6]. PPARγ is considered the master regulator of adipocyte differentiation and drives the expression of adipocyte-specific genes. The activation of PPARγ is necessary for the expression of adipocyte-specific genes, such as adipocyte protein 2 (aP2), lipoprotein lipase (LPL), and adiponectin and fatty acid synthase (FAS), which facilitates the cytoplasmic storage of massive amounts of triglycerides [7].

The phosphatidylinositol 3-kinase (PI3-K)/Akt signaling pathway plays a key role in regulating adipocyte differentiation and adipogenesis [8], [9]. The enforced expression of the constitutively active form of Akt increases glucose uptake and promotes spontaneous adipocyte differentiation in 3T3-L1 adipocytes [10]. In contrast, the RNAi-mediated decrease in Akt expression blocked the differentiation of 3T3-L1 cells [11], and mouse embryonic fibroblasts (MEFs) lacking Akt display an inability to differentiate into adipocytes and reduced body fat [12], [13]. These results highlight the pivotal role of Akt in adipogenic regulation in adipose tissue.

Akt phosphorylates and regulates a number of substrates involved in a diverse array of biological processes [14], many of which can enhance adipogenesis. Moreover, the Akt signaling pathway regulates the expression of C/EBPα and PPARγ during adipogenesis in 3T3-L1 cells [15], and 3T3-L1 cells lacking Akt could not induce PPARγ expression at the initiation of the adipogenesis program. The overexpression of PPARγ in Akt-deficient MEFs rescued the severe adipogenesis defect [12]. Interestingly, insulin signaling activates Akt through PI3K and induces the serine/threonine phosphorylation of the downstream target Glycogen synthase kinase 3 beta (GSK3β), which is involved in adipocyte differentiation of preadipose cells [16], [17]. Additionally, GSK3β is a critically important protein kinase in adipocyte differentiation as it phosphorylates a number of substrates, including the transcription factors β-catenin, C/EBPβ, C/EBPα, and glycogen synthase (GS).

Mushrooms and their components have been reported to have numerous positive health benefits, mainly on the basis of *in vitro* and *in vivo* animal trials. *Coprinus comatus* (CC), a novel cultivated edible mushroom found in Korea, has immense potential as a source of valuable medicinal compounds. Many physiological functions of CC have been reported, such as hypoglycemic, immunomodulation, hypolipidemic, antitumor and antibacterial effects [18], [19]. Mycelia extracts of CC have been shown to exhibit antioxidant potential [20] and hypoglycemic effects, as well as improved glucose tolerance [21]. Moreover, CC extracts exhibited hypolipidemic effects and antioxidant properties in diabetic mice, suggesting that their antioxidant activity could be directly or indirectly responsible for its hypoglycemic and hypolipidemic properties [22].

Many species of mushrooms have been investigated for their use as potential anti-obesity agents, and understanding how CC extracts function during adipogenesis is essential to developing new treatments for obesity. However, there have been few studies on the role of *Coprinus comatus* cap (CCC) in the regulation of adipogenesis and obesity. In the present study, we investigated the inhibitory effect of CCC on adipocyte differentiation in 3T3-L1 cells and its anti-obesity effects in high fat diet (HFD)-induced obese rats.

## Materials and Methods

### Preparation of *Coprinus comatus* cap extracts (CCCE)

Fresh fruit bodies of *Coprinus comatus* (CC) were obtained from the Bio Company (Moonsan, Korea). The caps of the fruit bodies were peeled off and air-dried in an oven, which began at 30°C, then increased 5°C every 3 h until it reached 40°C. The dried cap was milled into a powder (40 mesh). The *Coprinus comatus* caps (CCC) (15 g) were ground in an 80% (v/v) ethanol solution using a mixer, followed by extraction of the samples for 3 days with vigorous shaking at room temperature, and filtering through Whatman No. 1 filter paper. The ethanolic extracts of the *Coprinus comatus* caps (CCC) were concentrated using rotary-vacuum evaporation at 50°C and then freeze-dried.

### Measurement of total phenolic content using Folin-Ciocalteu assay

The total phenolic content of the CCCE was determined using a spectrophotometer according to the Folin-Ciocalteu colorimetric method [23]. Because quercetin is one of the polyphenol compounds found in CCC, the total phenolic content of ethanol extract of CCC was expressed as mg quercetin (Sigma-Aldrich, USA) equivalents (QE)/g.

### Measurement of total flavonoids

The total flavonoid content was determined as previously described [24] with slight modifications. Briefly, 0.25 mL of CCCE (100 µg/mL) was added to a tube containing 1 mL of double-distilled water. Next, 0.075 mL of 5% NaNO_2_, 0.075 mL of 10% AlCl_3_ and 0.5 mL of 1 M NaOH were added sequentially at 0, 5 and 6 min. Finally, the volume of the reacting solution was adjusted to 2.5 mL with double-distilled water. The solution had an absorbance of 410 nm that was detected using an Ultrospec 2100 Pro Spectrophotometer (Section 3.3). The results were expressed in mg quercetin equivalents (QE)/g.

### Measurement of free radical scavenging activity using DPPH assay

The free radical scavenging activity of CCCE (100 µg/mL in DW) was measured using the method of Brand-Williams [25], with some modification. The inhibition percentage was calculated using the following equation: Inhibition %  =  [(absorbance of control-absorbance of sample)/absorbance of control]×100. The absorbance was measured using a spectrophotometer (Ultrospec 2100 pro; Amersham Pharmacia Biotech Co., Piscataway, NJ, USA).

### Measurement of superoxide anion (O2^•−^) radical scavenging and hydroxyl (OH^•^) radical scavenging activity

Superoxide radicals were generated according to a method described in a previous paper [26]. The samples (100 µg/mL in DMSO) were added to the reaction solution containing 100 µL of 30 mM EDTA (pH 7.4), 10 µL of 30 mM hypoxanthine in 50 mM NaOH, and 200 µL of 1.42 mM nitroblue tetrazolium (NBT). After the solution was preincubated at room temperature for 3 min, 100 µL of 0.5 U/mL xanthine oxidase was added to the mixture, and the volume was brought up to 3 mL with 50 mM phosphate buffer (pH 7.4). After the solution was incubated at room temperature for 20 min, the absorbance was measured at 560 nm. The reaction mixture without xanthine oxidase was used as a blank (A1). The samples (A2) were added to the reaction mixture, in which O2^•−^ was scavenged, thereby inhibiting the reduction of NBT. The absorbance was measured, and the decrease in O2^•−^ was represented by A2-A1. The scavenging activity on the superoxide anion radical (SRSA) was calculated using the following equation: SRSA %  =  (A2 − A1/A1) ×100. The scavenging activity of the samples (100 µg/mL) in DMSO on the hydroxyl radical (OH^•^) was measured using the deoxyribose method [27] with a slight modification. The deoxyribose assay was performed in 10 mM phosphate buffer (pH 7.4) containing 2.5 mM deoxyribose, 1.5 mM H_2_O_2_, 100 µM FeCl_3_, 104 µM EDTA, and the test sample (0.5 mg/mL). The reaction was started by adding ascorbic acid to a final concentration of 100 µM. The reaction mixture was incubated for 1 h at 37°C in a water-bath. After incubation, the color was developed by the addition of 0.5% thiobarbituric acid followed by addition of ice-cold 2.8% trichloroacetic acid in 25 mM NaOH and incubation for 30 min at 80°C. A control was performed without the samples (A1). The samples (A2) were cooled on ice, and the absorbance was measured at 532 nm. The hydroxyl radical scavenging activity (HRSA) was calculated using the following equation: HRSA%  =  (A1− A2/A1) ×100.

### Cell culture

Mouse 3T3-L1 preadipocytes were purchased from the Korean Cell Line Bank (Seoul, Korea) and cultured as described elsewhere [5]. In brief, cells were cultured in Dulbecco's modified eagle high-glucose medium (DMEM) supplemented with 10% calf serum at 37°C in a humidified atmosphere of 5% CO_2_. At 1 day postconfluence (designated “day 0”), cell differentiation was induced with a mixture (DMI) of 0.5 mM 3-isobutyl-1-methylxanthine, 100 µM indomethacin, 0.25 µM dexamethasone and 167 nM insulin in DMEM containing 10% FBS. The 3-isobutyl-1-methylxanthine (MIX), dexamethasone (DEX), indomethacin, and Oil-Red O were obtained from Sigma-Aldrich (St. Louis, MO, USA). The medium was changed every 2 days. CCCs were added to the culture medium of the adipocytes on day 0. The cells were treated with 0, 40, or 150 µg/mL CCC extracts every day. After treatment with CCC for 3, 5 and 7 days, the 3T3-L1 adipocytes were lysed for western blot analysis. To analyze cell viability, the cytotoxicity of the CCC was evaluated using 3-(4, 5-demethylthiazol-2-yl)-2, 5-diphenyltetrazolium bromide (MTT). A549 human lung carcinoma cells obtained from the Korean Cell Line Bank (Seoul, Korea) were cultured in RPMI 1640 medium supplemented with 10% FBS and 1% penicillin/streptomycin in a 5% CO_2_ atmosphere at 37°C. Cells grown to 70–80% confluency were untreated (control) or treated with 40 or 150 µg/ml CCC in the absence or presence of insulin (50 ng/ml) or rosiglitazone (10 µM) in complete growth medium.

### Oil-Red O staining and triglyceride assay

For Oil-Red O staining, the cells were gently washed with phosphate-buffered saline (PBS) and stained with filtered Oil-Red O solution (60% isopropanol and 40% water) for 30 min. After staining, the Oil-Red O staining solution was removed, and the plates were rinsed with water and dried. The stained lipid droplets were viewed on an Olympus microscope (Tokyo, Japan). To analyze the content of the cellular triglycerides, the cells were washed with PBS, scraped into 200 µL of PBS and sonicated for 1 min. When the elective PI3K inhibitor LY294002 (Sigma-Aldrich, St. Louis, MO, USA) was used, the 3T3-L1 cells were incubated with or without 10 µM LY294002 in the presence or absence of CCC for 6 days. The lysates were assayed for their total triglyceride content using assay kits from Sigma-Aldrich (St. Louis, MO, USA) and for cellular protein using the Bio-Rad protein assay (CA, USA). The results were expressed as mg of triglyceride per mg of cellular protein.

### RT-PCR

Total RNA was isolated from 3T3-L1 adipocytes and A549 human lung cells using the Trizol reagent (Invitrogen, CA, USA). One microgram of total RNA was used for first strand cDNA synthesis with oligo (deoxythymidine) primers and Superscript II reverse transcriptase (Invitrogen, CA, USA). The target cDNA was amplified using the following primers: C/EBPβ, 5′-GACTACGCAACACACGTGTAACT-3′ and 5′-CAAAACCAAAAACATCAACAACCC-3′; C/EBPδ, 5′-GATCTGCACGGCCTGTTGTA-3′ and 5′-CTCCACTGCCCACCTGTCA-3′; PPARγ, 5′-TTTTCAAGGGTGCCAGTTTC-3′ and 5′-AATCCTTGGCCCTCTGAGAT-3′; C/EBPα, 5′-TTACAACAGGCCAGGTTTCC-3′ and 5′-GGCTGGCGACATACAGATCA-3′; LPL, 5-TCCTCTGACATTTGCAGGTCTATC-3′ and 5′-GTGAATCCAGTTATGGGTTCCAC-3; aP2, 5-AACACCGAGATTTCCTTCAA-3′ and 5′-TCACGCCTTTCATAACACAT-3; adiponectin, 5′-ACCTACGACCAGTATCAGGAAAAG-3′ and 5′-ACTAAGCTGAAAGTGTGTCGACTG-3′; β-actin (control), 5′-GACAACGGCTCCGGCATGTGCAAAG-3′ and 5′-TTCACGGTTGGCCTTAGGGTTCAG-3′. The amplification cycles included denaturation at 95°C for 50 sec, annealing at 55°C for 1 min and elongation at 72°C for 50 sec. After 30 cycles, the PCR products were separated by electrophoresis on a 1.5% agarose gel for 30 min at 100 V. The gels were stained with 1 mg/ml ethidium bromide and visualized with UV light using the BIO-RAD Gel Doc image analysis software (BIO-RAD Laboratories Inc., CA, USA).

### Luciferase reporter activity assay

The plasmids encoding PPARγ and PPARγ-Luc were kindly provided by Dr. Jae Bum Kim of Seoul National University. The control plasmid encoding *Renilla* luciferase and the Dual-luciferase assay kit were purchased from Promega (www.promega.com). Because CHO cells do not express PPARγ, a mixture containing the PPARγ, RXRα, PPARγ-Luc, and *Renilla*-Luc plasmids were co-transfected into the CHO cells using the Lipofectamine 2000 reagent (Invitrogen, USA) according to the manufacturer's instruction. After transfection, the cells were treated with 100 µM rosiglitazone (PPARγ agonist) in the absence or presence of CCC for 24 h. The post-transfection luciferase activities were measured using the Dual-Luciferase Reporter Assay System (Promega, Madison, WI) using a GloMax20/20 luminometer (Turner Biosystems, Sunnyvale, CA). The results were normalized to the *Renilla* luciferase activity.

### Measurement of Glucose uptake

Glucose uptake activity was measured using a fluorescent D-glucose analogue 2-[N-(7-nitrobenz-2-oxa-1,3-diazol-4-yl)amino]-2-deoxy-D-glucose (2-NBDG) (Invitrogen, Carlsbad, USA) in 3T3-L1 cells as a previously described method with slight modifications [28]. Briefly, 3T3-L1 preadipocytes were differentiated with various concentrations (10, 40, and 150 µg/ml) of CCC or DMSO for 6 days in 6 well plates. After washing, differentiated 3T3-L1 cells were treated with or without a given concentrations of CCC and Insulin in the absence or presence of 10 µM 2-NBDG for 2 h. Then, cells were washed twice with PBS, the fluorescence intensity of cellular 2-NBDG in each well was measured at an excitation wavelength of 485 nm and an emission wavelength of 530 nm using Fluorescent microplate reader (Molecular Devices, USA).

### Western blot analysis

Western blot analysis was performed according to standard procedures. Briefly, 3T3-L1 cells, A549 human lung cells, and epididymal fat pads were homogenized in lysis buffer containing 50 mM Tris-HCl (pH 8.0), 0.4% Nonidet P-40, 120 mM NaCl, 1.5 mM MgCl_2_, 0.1% SDS, 2 mM phenylmethylsulfonyl fluoride, 80 µg/mL leupeptin, 3 mM NaF and 1 mM DTT. The cell lysates were separated by 10% SDS-polyacrylamide gel electrophoresis, transferred onto a polyvinylidene fluoride membrane (Amersham Pharmacia, England, UK), blocked with 5% skim milk and hybridized with primary antibodies. The PPARγ, C/EBPβ, C/EBPα, aP2, Akt, and GSK3β antibodies were purchased from Cell Signaling, and the monoclonal β-actin antibody was purchased from Chemicon. The HRP-labeled mouse anti-rabbit IgG antibody was purchased from Jackson ImmunoResearch. The chemiluminescence kit was purchased from Pierce (Rockford, IL). After incubation with horseradish peroxidase-conjugated secondary antibody at room temperature, the immunoreactive proteins were detected using a chemiluminescent ECL assay kit (Amersham Pharmacia, UK) according to the manufacturer's instructions.

#### Animal experiments

The study protocol was approved by the Animal Care and Use committee of Gyeongsang National University (Approval Number: GNU-121213-R0049). Five-week-old male Sprague-Dawley (SD) rats, weighing approximately 160 g, were purchased from the Central Lab. Animal Inc. (Seoul, Korea). All rats were housed in polycarbonate cages in a room maintained at 22°C and 55% relative humidity. The room was exposed to alternating 12 h periods of light and dark. The rats were randomly divided into the following 4 groups: group fed a regular diet (RD, n = 10); group fed a high-fat diet (HFD, rodent diet with 60% kcal fat, Research Diet, Korea); and treatment groups fed a high-fat diet plus CCC at 60 mg/kg (CCC 60) or 200 mg/kg (CCC 200). All of the rats were allowed free access to food and water for 5 weeks. Food intake was measured daily, and the rats were weighed every two days. Obese rats were generated by feeding the rats a high-fat diet (HFD).

### Biochemical assays

After 5 weeks on experimental diets, the rats were euthanized, and the tissues were dissected out and analyzed. The body and fatty tissue weights were measured with sensitivity limits of 0.1 g and 0.01 g, respectively. The body mass index was calculated by dividing the weight (g) by the square of the body length (cm^2^). Blood was collected from each rat, stored at 37°C for 30 min, and centrifuged at 4000 g at 4°C for 10 min to obtain the plasma. The epididymal fat pad and perirenal fat pad were excised, weighed and stored at −20°C until assayed. The concentrations of plasma triglycerides (TG), total cholesterol (TC), and high-density lipoprotein (HDL)-cholesterol were assayed enzymatically using commercial kits (Asan phams, Co., Korea).

### Histological Analysis

The epididymal fat pads and liver tissues was removed and fixed in 10% neutral buffered formalin. The fat pads and livers were subsequently embedded in paraffin, sectioned into 5 µm sections (Leica, Wetzlar, Germany), and stained with hematoxylin-eosin for microscopic assessment (Olympus, Tokyo, Japan). Three different cross-sectional areas and their cell populations were calculated using an image analysis program (Image-Pro Plus 6.0).

### Statistical analysis

The data are expressed as the mean ± SD. The significant differences in the treatment means were determined using ANOVA and Duncan's multiple range tests at p<0.05.

## Results

### Total phenol content (TPC) and total flavonoid content (TFC) of CCCE

The TPC and TFC contents of the CCCE were found to be 15.5±1.23 mg quercetin equivalent/g, and 3.34±0.67 mg quercetin equivalent/g extract, respectively (Table 1).

**Table 1:** Antioxidant capacities, total phenolic and flavonoids content of the CCC extracts.

	DPPH^a^	HRSA^b^	SRSA^c^	TPC^d^ (mgQE/g)	Flavonoid (mgQE/g)
CCC	12.3±0.85^y^	14.2±0.78^z^	43.3±1.4^z^	15.5±1.23^y^	3.3±0.67^x^

a DPPH, DPPH radical scavenging activity;

b HRSA, hydroxyl radial scavenging activity;

c SRSA, superoxide anion radical scavenging activity;

d TPC, total phenolic acid. Total phenolic acid and total flavonoid content are expressed as milligrams of quercetin equivalent (QE)/g of extract.

x–z The values are presented as the mean ± SD. P<0.01 represents a significant difference between the samples (n = 4).

### DPPH, hydroxyl, and superoxide anion radical scavenging activity

The antioxidant properties were summarized in Table 1. The CCCEs exhibited potent DPPH radical scavenging activity. The hydroxyl radical scavenging activity of the CCCEs was assessed by the reduction of nitroblue tetrazolium, and the CCCEs inhibited hydroxyl radical generation. The extract was found to possess potent antioxidant activity against superoxide radicals.

### CCC extract inhibits lipid accumulation during the differentiation of 3T3-L1 preadipocytes

**Figure 1:**
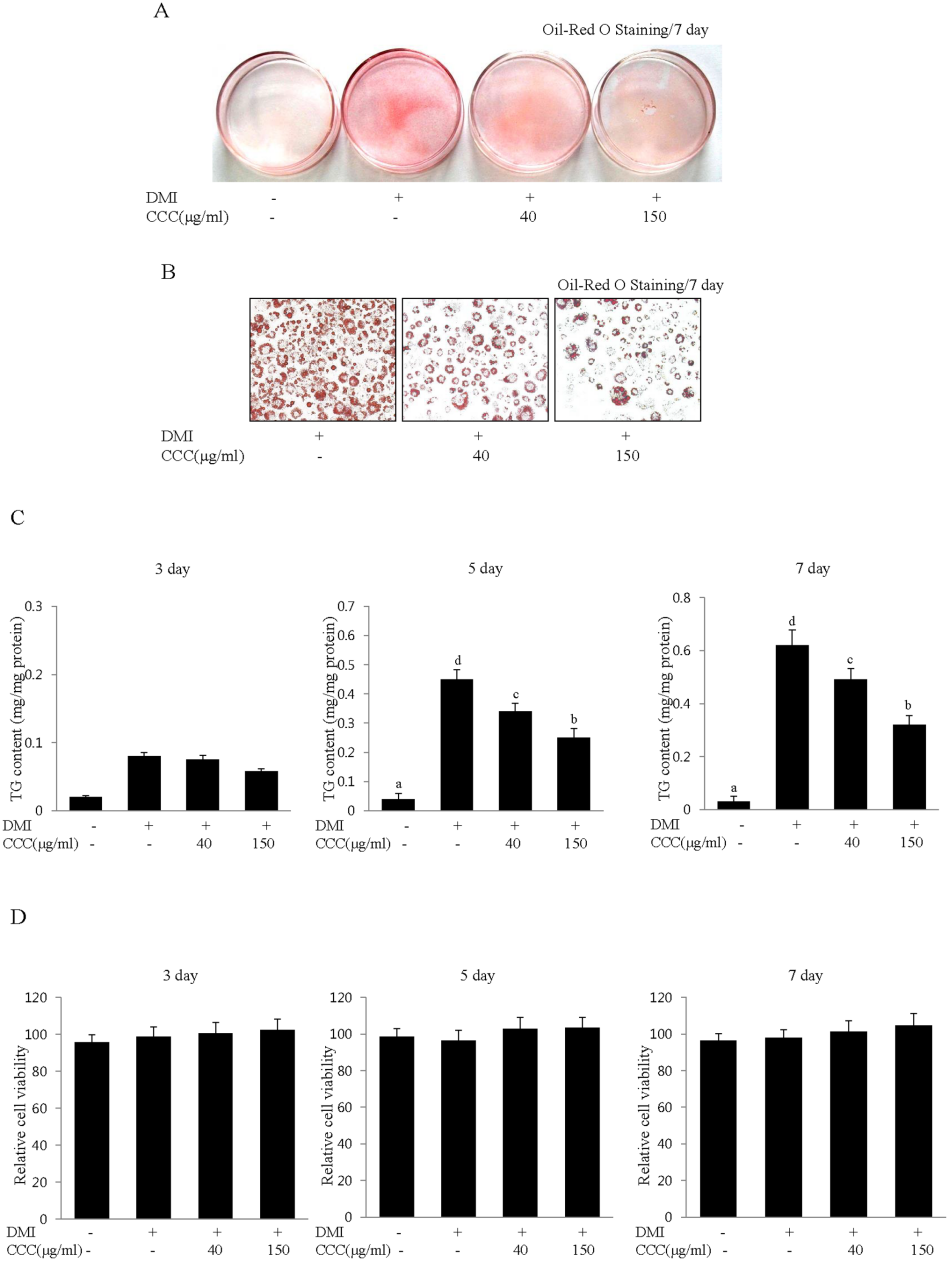
CCC inhibits lipid accumulation during the differentiation of 3T3-L1 preadipocytes. (A) Insulin-induced differentiation of 3T3-L1 adipocytes was repressed by CCC. Confluent 3T3-L1 preadipocytes were differentiated into adipocytes in DMI medium containing different concentrations (0, 40, or 150 µg/ml) of CCC for 7 days (from day 0 to 7). (A) The insulin-induced differentiation of 3T3-L1 adipocytes was repressed by CCC treatment. Oil-Red O staining was performed on day 7 after the induction of differentiation. DMI: Adipogenic differentiation medium (0.5 mM 3-IBMX, 100 µM indomethacin, 0.25 µM dexamethasone and 167 nM insulin). CCC: *Coprinus comatus* cap extract. (B) Microscopic observations of the Oil-Red O staining showed a gradual reduction in the lipid content of the 3T3-L1 adipocytes after CCC treatment. (C) Effects of CCC extract on intracellular triglyceride accumulation in 3T3-L1 adipocytes. The triglyceride content was significantly reduced by CCC treatment on days 3, 5, and 7 after the induction of differentiation in 3T3-L1 cells. The data shown are representative of at least three independent experiments. The values are presented as the mean ± SD. The bars with different letters are significantly different (*p<0.05) as determined by Duncan's multiple range test. (D) Effects of CCC on cell cytotoxicity in 3T3-L1 adipocytes. The cells were incubated for 7 days with various concentrations (0, 40, or 150 µg/ml) of CCC after the induction of differentiation. Cell viability was measured on days 3, 5, and 7 using an MTT assay. The results were confirmed by three independent experiments, which were each conducted in triplicate. The data are presented as the mean ± SD.

To examine the anti-adipogenic effect of CCC on the differentiation of preadipocytes into adipocytes, 3T3-L1 preadipocytes were treated with DMI in the presence or absence of 40 or 150 µg/mL CCC for 7 days. The 3T3-L1 preadipocytes were fully differentiated at 7 days after induction with the DMI mixtures. Lipid accumulation, as a major marker of adipogenesis, was quantified at day 7 by Oil-Red O staining. The triglycerides from the fully differentiated adipocytes on day 7 were stained with Oil-Red O staining solution, which stains lipid droplets to indicate lipid accumulation. The cells treated with lower concentrations of CCC (40 µg/mL) showed a high level of lipid droplet staining, whereas the cells incubated with 150 µg/mL CCC exhibited markedly reduced lipid staining on differentiation day 7 (Fig. 1A). Microscopic observations of the Oil-Red O staining showed gradual reductions in the amounts of lipid droplets with increasing concentrations of CCC in a dose-dependent manner, demonstrating that treatment with CCC attenuated lipid accumulation in the 3T3-L1 adipocytes (Fig. 1B). Similar effects on lipid accumulation with the same treatment were observed by measuring the intracellular triglyceride (TG) content on days 3, 5 and 7 of the differentiation period in 3T3-L1 cells. The results show that treatment with CCC reduced the triglyceride content and that the inhibition of intracellular TG in 3T3-L1 adipocytes occurred in a dose-dependent manner and was dramatically reduced by 21% and 43% at concentrations of 40 and 150 µg/mL of CCC on day 7, respectively (Fig. 1C).

To examine the cytotoxic effects of CCC on the differentiation of 3T3-L1 preadipocytes, we induced 3T3-L1 cells to differentiate by treating them with DMI mixtures every 2 days in the presence or absence of CCCE. The cells were treated for 7 days with various concentrations of CCC, ranging from 0 to 150 µg/mL, and were then subjected to MTT assays on days 3, 5 and 7 after the initiation of differentiation. The results of the MTT assay showed that concentrations of up to 150 µg/mL CCC did not decrease the cell viability (Fig. 1D). The CCC treatments had no effect on cell cytotoxicity up to 150 µg/mL (Fig. 1D). Taken together, these results clearly indicated that the inhibitory effects of CCC on lipid droplet formation and triglyceride accumulation were not due to cytotoxicity. From these results, we elucidated that CCC inhibited adipocyte differentiation in 3T3-L1 cells.

### CCC inhibits the mRNA and protein expression of transcription factors and adipocyte markers during adipogenesis

**Figure 2:**
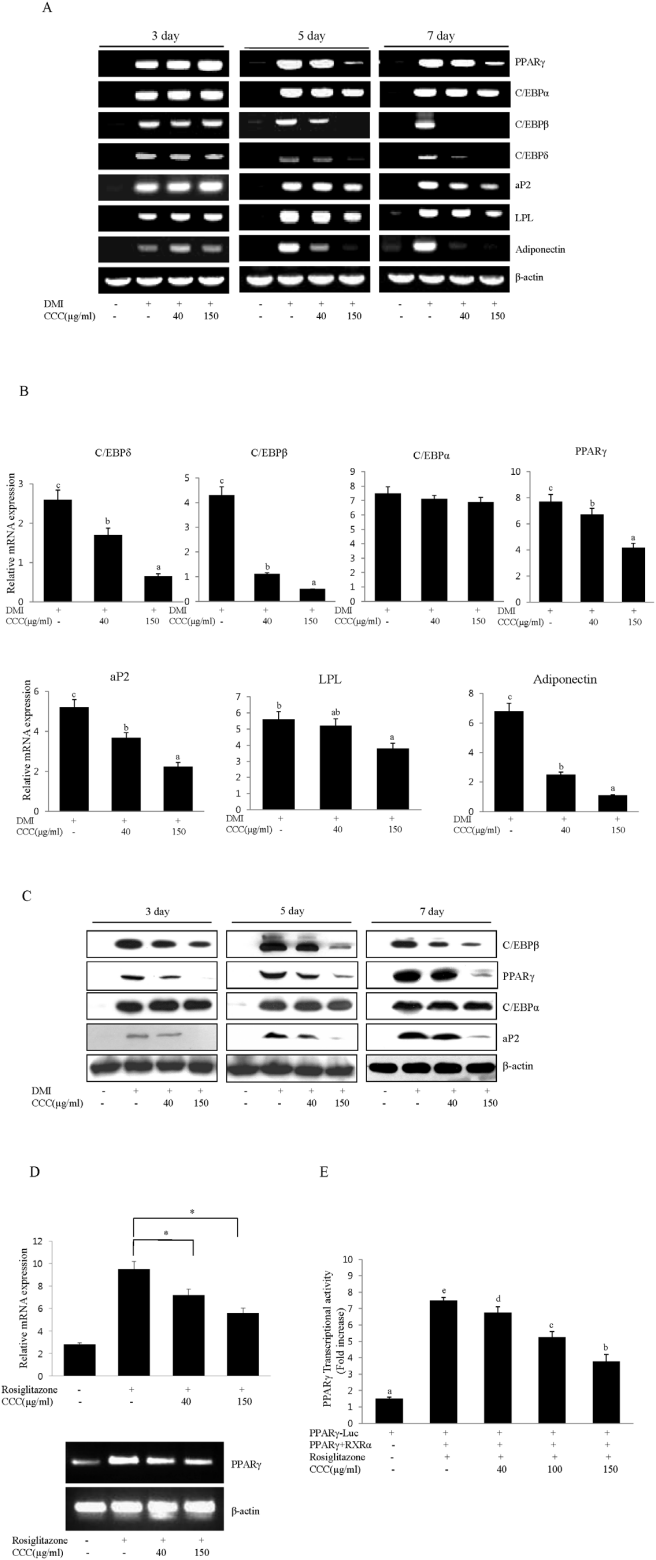
CCC down-regulated the expression of the main adipogenesis and adipocyte-specific transcription factors during adipocyte differentiation in 3T3-L1 preadipocytes. (A) Effects of CCC on PPARγ, C/EBPα, C/EBPβ, C/EBPδ, aP2, LPL, and adiponectin mRNA expression in 3T3-L1 cells. Confluent 3T3-L1 preadipocytes were induced to differentiate in the presence of different concentrations of CCC (from 0 to 150 µg/ml) for 7 days. Total RNA was isolated from 3T3-L1 adipocytes on days 3, 5 or 7 after the induction of differentiation, and gene expression analysis was performed by RT-PCR. The amplification of β-actin was performed as a loading control. All of the experiments were performed in three independent experiments. (B) The expression levels of adipogenesis and adipocyte-specific genes were determined during adipocyte differentiation at day 7. The mRNA expression of PPARγ, C/EBPα, C/EBPβ, C/EBPδ, aP2, and LPL were normalized using β-actin as a control. The bars with different letters are significantly different (*p<0.05) as determined by Duncan's multiple range test. (C) CCC reduced the protein levels of PPARγ, C/EBPβ, and aP2 genes in 3T3-L1 adipocytes. Total protein was isolated from 3T3-L1 adipocytes on days 3, 5 or 7 after the induction of differentiation. Total cellular proteins were immunoblotted for PPARγ, C/EBPβ, aP2, and β-actin, as indicated. Similar results were obtained from 3 replicates. (D) Effect of CCC on PPARγ mRNA expression in A549 lung cancer cells. A549 lung cancer cells were treated with 40 or 150 µg/ml CCC in the absence or presence of rosiglitazone (10 µM) for 24 h in complete growth medium. Total RNA isolated from A549 cells was subjected to RT-PCR, and all of the gene transcripts were normalized using β-actin as a control. The data represented the mean ± SD of 3 different experiments. *p<0.05, **P<0.01. (E) PPARγ and RXRα were cotransfected with the PPARγ-Luc reporter construct into CHO cells for 24 h. The cells were treated with 100 µM rosiglitazone in the absence or presence of CCC. After 24 h, luciferase activity was assayed. The data are the mean ± SD values of at least three independent experiments. The bars with different letters are significantly different (*p<0.05) as determined by Duncan's multiple range test.

Adipogenesis is accompanied by increased expression of adipogenic transcription factors and adipocyte-specific genes. To examine the effect of CCC on the expression of adipogenic transcription factors, mRNA expression levels of PPARγ and the C/EBP family were measured during the differentiation of 3T3-L1 cells in the presence or absence of CCC. On days 3, 5, and 7, we examined the expression of the adipocyte-specific transcription factors, C/EBPβ, C/EBPδ, PPARγ and C/EBPα. The mRNA levels of C/EBPβ, δ, α and PPARγ were significantly increased following induction of adipogenesis on days 3, 5, and 7; however, treatment with CCC reduced the mRNA levels of C/EBPβ, δ, and PPARγ in a time- and concentration-dependent manner (Fig. 2A and B). Western blot analysis confirmed that the expression of C/EBPβ and PPARγ was significantly down-regulated in the presence of 150 µg/ml of CCC extract compared to the control adipocytes that were induced by the DMI mixtures (Fig. 2C). In addition, CCC also inhibited PPARγ mRNA expression in A549 lung cancer cells (Fig. 2D), indicating that CCC affected the expression of PPARγ in DMI-independent manner. RT-PCR analysis showed that the mRNA expression levels of the genes controlling adipogenesis and adipocyte fatty acid metabolism, including lipoprotein lipase (LPL), fatty acid binding protein (aP2), and adiponectin, were also significantly increased after the induction of adipogenesis in 3T3-L1 cells; however, the presence of CCC resulted in the down-regulation of LPL, aP2, and adiponectin expression in dose-dependent manner compared to the differentiated adipocytes (Fig. 2). The patterns of adipogenic protein expression were similar to the patterns of adipogenic mRNA expression. We further investigated whether the reduction of PPARγ by CCC regulated the expression of its target gene, aP2. The treatment of CCC significantly inhibited the expression of aP2 in a dose-dependent manner during the differentiation of 3T3-L1 cells (Fig. 2C). Intriguingly, although the expression of PPARγ was reduced in the presence of CCC, there was no significant change in C/EBPα mRNA or protein expression compared to the control cells that were differentiated with the DMI mixtures (Fig. 2A and C). These results suggest that CCC obviously inhibited lipogenesis and adipogenesis through the down-regulation of the key adipogenic genes, C/EBPβ, C/EBPδ, PPARγ and aP2. Next, to further investigate whether CCC could inhibit the transcriptional activity of PPARγ, the PPARγ expression plasmid and the PPARγ luciferase reporter construct driven by a promoter containing three repeats of the PPARγ binding site were co-transfected into CHO cells. We found that CCC significantly suppressed the rosiglitazone-induced PPARγ transcriptional activity (Fig. 2E). Together, these results demonstrated that CCC prevented adipocyte differentiation through an antagonistic effect on PPARγ transcriptional activity.

### The Akt/GSK3β signaling pathway participated in the regulation of adipocyte differentiation by CCC in 3T3-L1 cells

**Figure 3:**
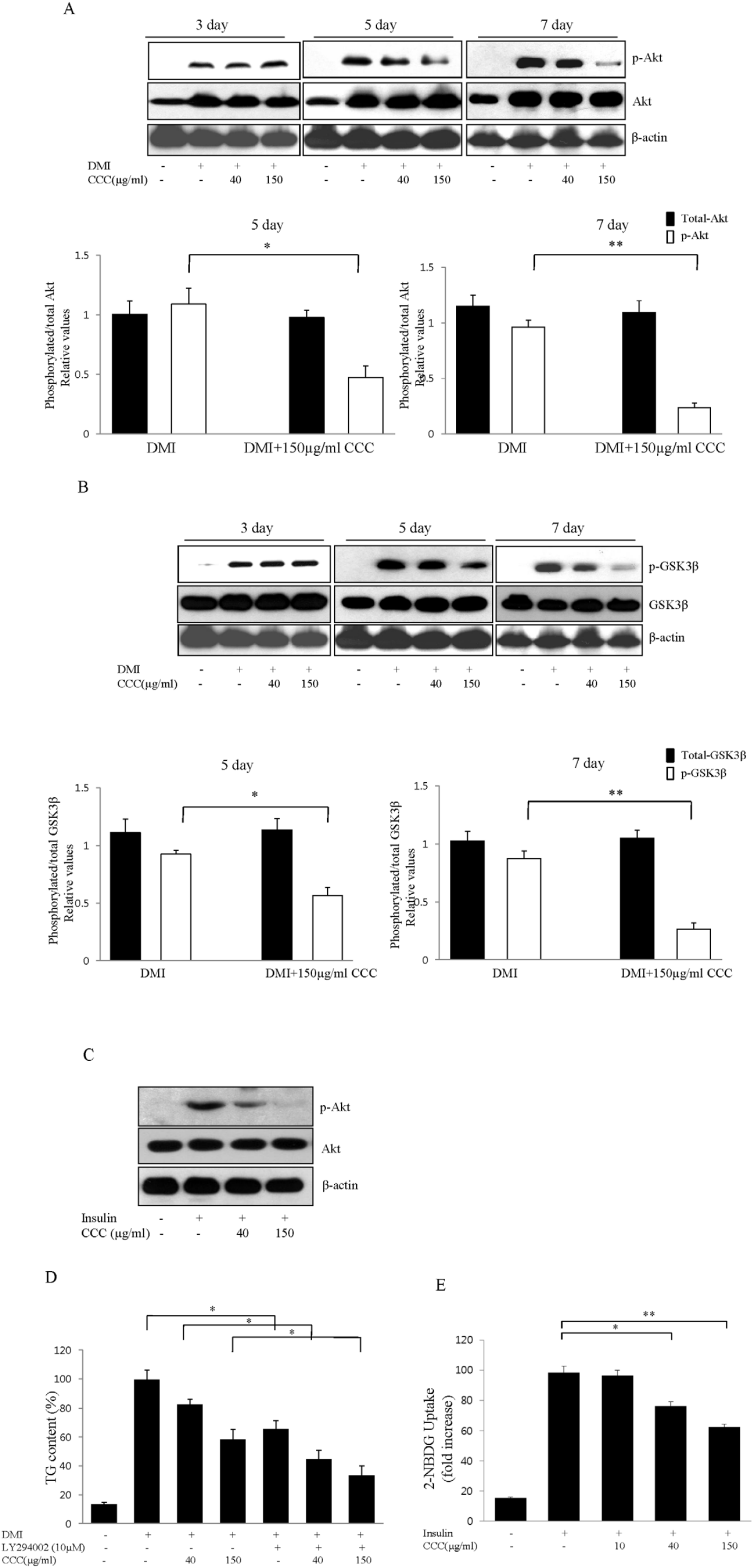
Effects of CCC on the regulation of Akt and GSK3β during adipocyte differentiation. Confluent 3T3-L preadipocytes were treated either with vehicle or CCC (0, 40, or 150 µg/ml) in the differentiation medium during day 0 to 7 of adipogenesis. (A) The protein level and the degree of Akt phosphorylation were analyzed in 3T3-L1 cells on days 3, 5, and 7 after the induction of differentiation. These experiments were conducted as independent experiments in triplicate. The data represent the mean ± SD. *p<0.05. **P<0.01. (B) Effect of CCC on GSK3β activation in 3T3-L1 adipocytes. 3T3-L1 adipocytes were treated with CCC extracts at the indicated concentrations, and the phosphorylation levels of GSK3β were determined by western blot analysis. The data are presented as the mean ± SD values of at least three independent experiments. *p<0.05. **P<0.01. (C) Effect of CCC on Akt phosphorylation in A549 lung cancer cells. A549 lung cancer cells were treated with 40 or 150 µg/ml CCC in the absence or presence of insulin (50 ng/ml) for 12 h. The representative Western bolts from one of three independent experiments were shown. (D) Effects of the PI3K/Akt inhibitor LY294002 on the CCC-induced inhibition of adipocyte differentiation in 3T3-L1 cells. 3T3-L1 cells were treated with CCC during differentiation in the presence or absence of 10 µM LY294002. The intracellular lipid accumulation was measured using a triglyceride assay. The data are expressed as the mean ± SD of three independent experiments. *P<0.05. (E) Effect of CCC on insulin-stimulated glucose uptake. Glucose uptake activity was analyzed by measuring of 2-NBDG in differentiated 3T3-L1 cells. 3T3-L1 cells were induced to differentiate into adipocytes for 6 days in DMI medium without or with CCC (10, 40, and 150 µg/m), and glucose uptake was then measured. The data are represented as the value relative to that of the undifferentiated cells. The data are presented as the mean ± SD from 3 independent experiments. *p<0.05. **P<0.01.

To determine whether CCC affects the Akt signaling pathway, 3T3-L1 adipocytes were treated with DMI in the presence or absence of CCC. *The insulin*-mediated serine phosphorylation (Ser473) *of Akt* was increased following the DMII-induced differentiation of 3T3-L1 cells. However, the treatment of 3T3-L1 cells with 40 µg/ml or 150 µg/ml of CCC decreased the levels of Akt phosphorylation on day 5. Moreover, the exposure of 3T3-L1 cells to 150 µg/ml CCC during the 7 day adipocyte differentiation period dramatically inhibited the increase in the serine phosphorylation of Akt caused by the insulin treatment (Fig. 3A). Because the insulin-induced Akt phosphorylation leads to the serine 9 phosphorylation (Ser9) of GSK3β, the levels of GSK3β serine phosphorylation were examined. The expression of wild type GSK3β was not altered by CCC treatment, whereas CCC treatment significantly decreased the amount of serine phosphorylation on GSK3β in a dose-dependent manner on days 5 and 7 of the 3T3-L1 cell differentiation period (Fig. 3B). To investigate whether CCC had a role in phosphorylating Akt at Ser473, we treated A549 lung cancer cells as shown in Fig. 3C and performed western blot analysis. In agreement with the effects of CCC in the 3T3-L1 adipocytes, CCC attenuated insulin-induced Akt phosphorylation in A549 lung cancer cells. Taken together, these results demonstrated that the activation of phosphorylated Akt was inhibited by CCC, which in turn suppressed the phosphorylation of its substrate kinase, GSK3β.

### CCC inhibited 3T3-L1 adipocyte differentiation through the Akt signaling pathway

To further investigate whether the PI3K/Akt signaling pathway was directly involved in the inhibition of adipocyte differentiation by CCC, we examined whether CCC acted through the PI3K/Akt pathway using LY294002, a specific inhibitor of PI3K/Akt. Following the induction of differentiation, 3T3-L1 cells were treated with either CCC alone or a combination of CCC and 10 µM LY294002 for 6 days. After treatment with an MDI mixture for 6 days, the differentiated 3T3-L1 cells had a much higher level of lipid droplets than the undifferentiated cells, as shown by the increase in intracellular triglyceride content (Fig. 3D). The degree of lipid accumulation decreased with increasing concentrations of CCC. Treatment with LY294002 significantly reduced the DMI-induced adipocyte differentiation of 3T3-L1 cells. Moreover, the triglyceride contents of 3T3-L1 adipocytes treated with a combination of 150 µg/mL CCC and LY29400 was 55% of the cultures that were treated with CCC alone (Fig. 3D). Thus, triglyceride accumulation was strongly inhibited in the presence of CCC, suggesting that CCC prevented adipocyte differentiation through the inhibition of the PI3K/Akt signaling pathway in 3T3-L1 cells.

### CCC reduced glucose uptake in 3T3-L1 adipocytes

We examined the effect of CCC on glucose uptake in 3T3-L1 adipocytes, using 2-NBDG. After reaching subconfluency, 3T3-L1 cells were incubated with differentiation medium in the presence of various concentrations (10, 40, and 150 µg/ml) of CCC for 6 days. CCC inhibited the glucose uptake in a concentration dependent manner and 35% reduction in insulin-stimulated glucose uptake was observed at a concentration of 150 µg/ml CCC (Fig. 3E).

### CCC reduced the body weight and fat mass in HF-induced obese rats

**Figure 4:**
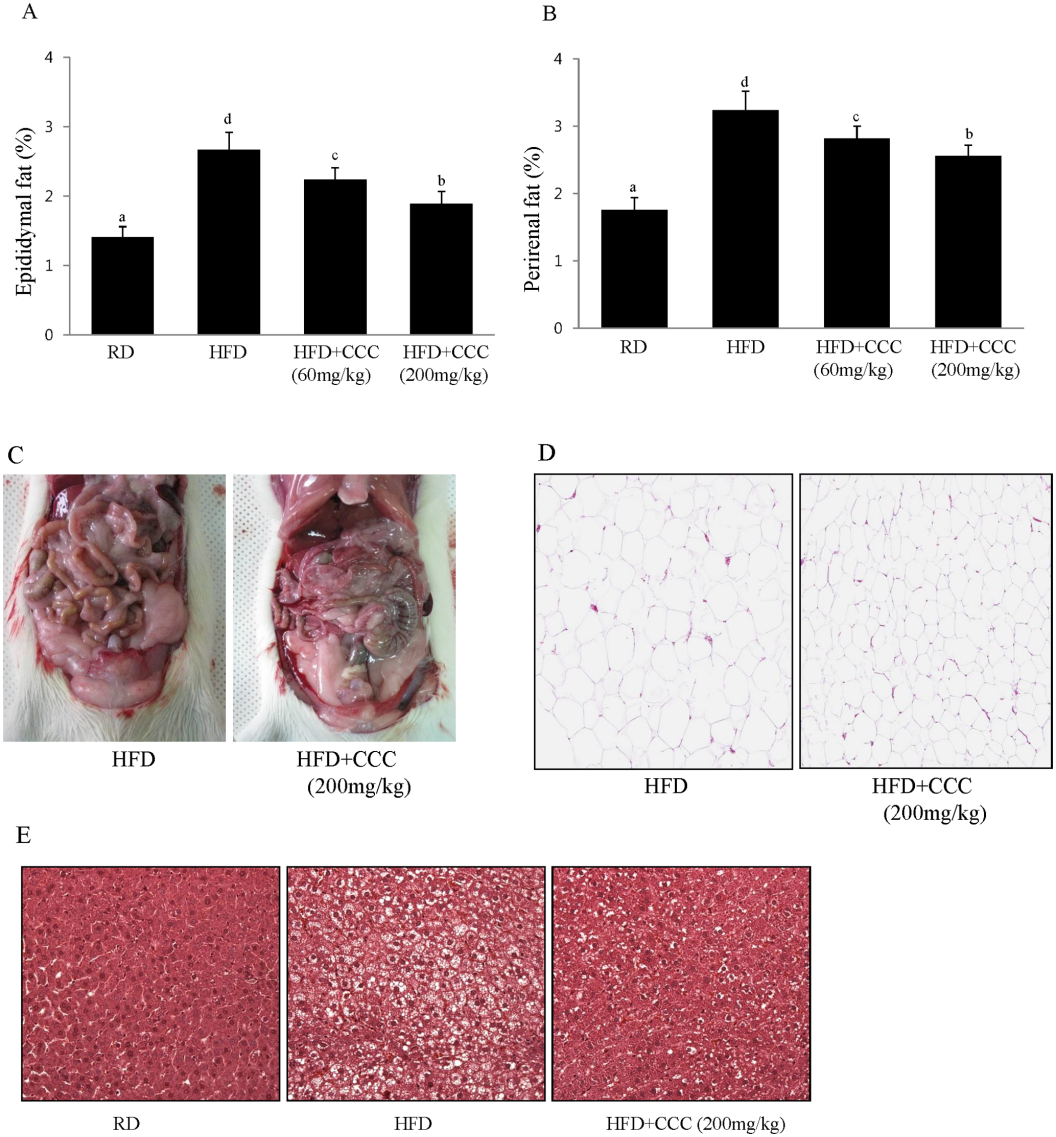
Treatment with CCC decreased the adipose tissue of the HFD-induced obese rats. The epididymal fat and perirenal fat weights were significantly decreased in the HDF+CCC (200 mg/kg) group compared to the HFD group. (A) Effects of CCC on epididymal fat weight. The weights of the epididymal fatty tissue were calculated by dividing the fatty tissue weight by the body weight (fatty tissue/body weight x 100). The values are expressed as the mean ± SD. The bars with different letters are significantly different (*p<0.05). (B) Effects of CCC on perirenal fat weight. The values are expressed as the mean ± SD. The bars showing different letters indicate significant differences among each group of bars, according to Duncan's test; *P<0.05. The means sharing a common letter do not significantly differ. (C) Morphology of the fat tissue altered by CCC in the HFD-induced obese rats. (D) Histological staining of epididymal adipose tissue. Epididymal adipose tissues were isolated and stained with hematoxylin and eosin (H&E), then examined microscopically. Representative micrographs showing the reduced adipocyte size in the HFD+CCC (200 mg/kg) group are shown. (E) Representative H&E-stained liver section. Liver tissues were isolated and stained with H&E, then examined microscopically. Representative micrographs showing the reduced number and size of lipid droplets in the HFD+CCC (200 mg/kg) group are shown.

Treatment with CCC inhibited adipogenesis and adipocyte differentiation in 3T3-L1 cells, suggesting that CCC attenuates obesity induced by a high-fat diet (HFD). To determine whether CC could regulate obesity, rats were fed a regular diet (RD), a high-fat diet (HFD), a high-fat diet supplemented with 60 mg/kg CCC (CCC 60), or a high-fat diet supplemented with 200 mg/kg CCC (CCC 200) for 5 weeks. The high fat diet significantly increased the rats' body weight after 5 weeks in the HFD group compared to the normal group (RD). This difference was present after the first week and was maintained until the completion of the study (Table 2). The body weight at week 5 was significantly reduced in the CCC 60 and CCC 200 groups compared to that of the HFD group (25% and 36%, respectively). Interestingly, despite no significant differences in the daily food intake between the HFD and HFD+ CCC groups, the CCC 200 group had a significantly lower body weight that the HFD group. Remarkably, the decreased weights of the epididymal and perirenal fat pads were also observed when the rats were fed a diet supplemented with CCC, indicating that CCC inhibited fat accumulation (Fig. 4A and B). Furthermore, the perirenal fat pad masses in the CCC groups were significantly reduced compared to the HFD group (Fig. 4C). The effect of CCC on the epididymal fat weight was determined by histological examination. The size of the adipocytes in the epididymal fat of the CCC 200 group was significantly smaller than in the HFD-induced obese rats (Fig. 4D). The addition of CCC to the HFD negated the effect of HFD on liver toxicity (data not shown).

**Table 2:** Effects of CCC extracts on the body weight of HFD-induced obese rats.

Groups	RD	HFD	HFD+CCC(60 mg/kg)	HFD+CCC(200 mg/kg)
Body weight gain (g/weeks)				
1st week	38.6±3.22	50.8±4.1**	46.7±2.56^a^	44.2±3.35^a^
2nd week	36.4±3.25	53.6±4.15**	48.5±3.87^a^	45.6±4.23^b^
3rd week	37.8±3.42	57.6±3.65**	50.6±4.52^a^	44.6±3.57^b^
4th week	39.6±4.33	60.5±4.55**	54.5±5.02^a^	46.8±3.98^b^
5th week	35.8±4.22	55.3±4.83**	49.5±4.36^a^	44.7±4.07^b^
6th week	34.5±3.48	56.5±3.25**	48.7±4.28^a^	43.6±3.18^b^

The results are presented as the mean ± SD. RD, regular diet-fed rat; HFD, high-fat diet-fed rat; HFD+CCC (60 mg/kg), high-fat diet containing 60 mg/kg BW CCC-fed rat; HFD+CCC (200 mg/kg), high-fat diet containing 200 mg/kg BW CCC-fed rat. The body weight was measured twice a week. Results are presented as the means ± SD.

a P<0.05,

b P<0.01 compared to the HFD group;

**P<0.01 compared to the RD group.

#### CCC inhibited HFD-induced fat accumulation in the liver

Haematoxylin and eosin staining was performed for analysis of the effect of CCC on HFD-induced lipid accumulation in the liver. Lipid accumulation was highly amplified in the HFD group compared to the normal group (RD). Histological analysis showed that HFD-fed rats developed hepatocellular micro- and macrovesicular vacuolation as a result of fat accumulation (Fig. 4E). However, the lipid accumulations induced by HFD were significantly reduced in the HFD+CCC group, indicating that CCC is capable of preventing lipid accumulation and hepatic steatosis in HFD-induced fatty liver. Additionally, treatment of CCC (200 mg/kg) caused a significant reduction in level of hepatic TBARS as compared to the HFD group, suggesting that CCC increased the antioxidant capacity in the liver of HFD-induced obese rat (data not shown).

### CCC reduced serum triglyceride (TG) and total-cholesterol (TC) levels in the HFD-induced obese rats

**Figure 5:**
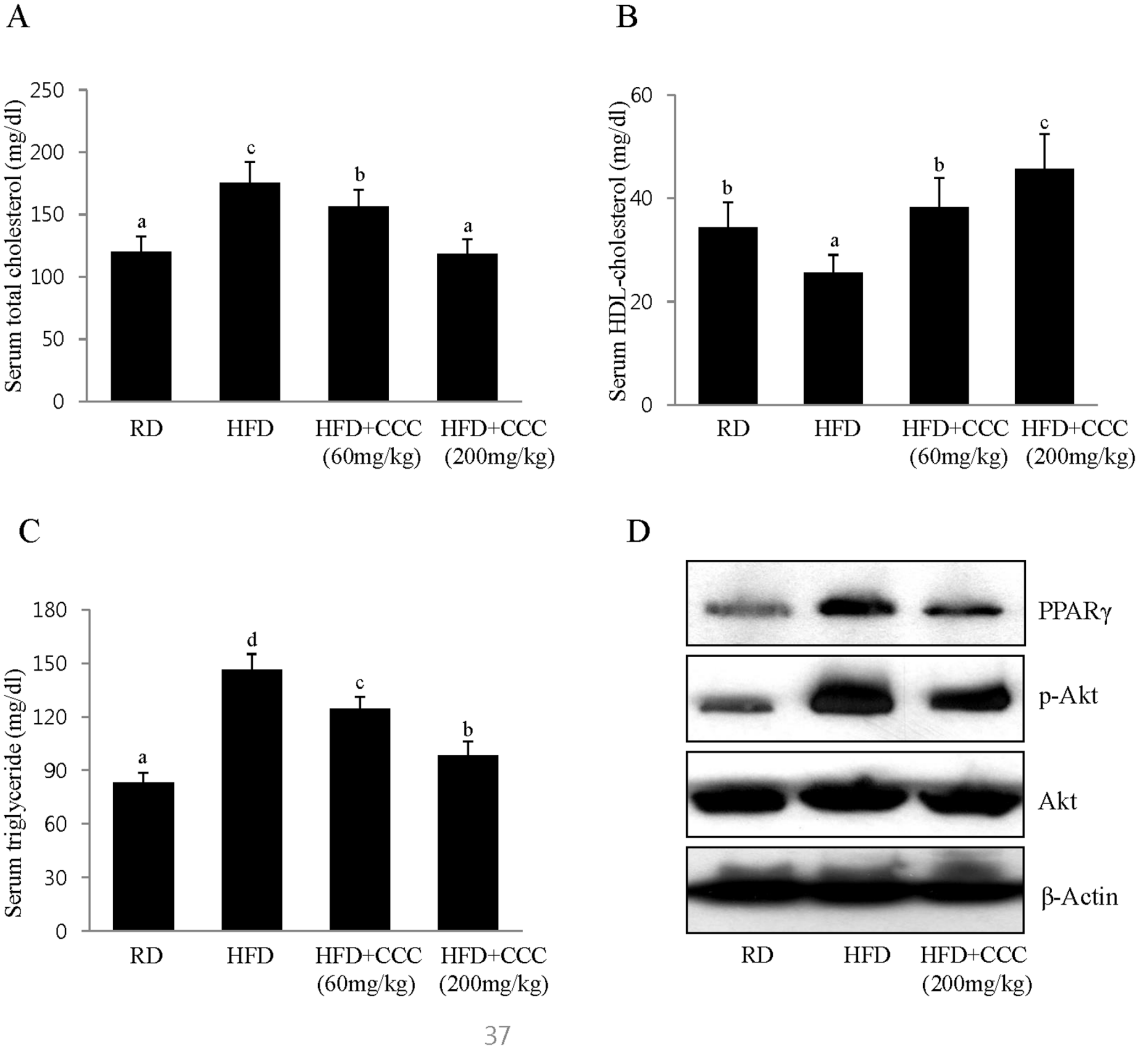
Effect of CCC on the lipid contents of the HFD-induced obese rats. (A, B, C) Significant decreases in the levels of serum triglycerides and total cholesterol were observed in the CCC-treated groups compared to the HFD-induced obese rats. HDL-cholesterol levels in the CCC groups were increased compared to the HFD group. The values are expressed as the mean ± SD. The bars showing different letters indicate significant differences among each group of bars, according to Duncan's test; *P<0.05. (D) CCC reduced the phosphorylation of Akt and the expression of PPARγ in the HFD-induced obese rats. PPARγ and phospho-Akt protein expression in the epididymal fat tissue was determined by western blot analysis.

To determine whether CCC has beneficial effects on the circulating lipid profiles, we examined its effects on serum triglyceride (TG) and total cholesterol (TC) levels. In a manner similar to the increases observed in body weight and fat mass, the serum levels of TG and TC in the HFD group were significantly increased compared to the regular diet group (RD) (Fig. 5A and C). However, treatment with CCC (CCC 200) caused a significant reduction in the serum TG and TC levels by 32% and 46%, respectively, compared with the rats that were given HFD alone. Moreover, the serum high-density lipoprotein (HDL) cholesterol levels were significantly increased in the CCC group compared to the HFD group (Fig. 5B). Thus, the CCC extracts significantly reduced the serum TG and TC levels and increased the HDL-cholesterol levels in a dose-dependent manner. These results indicated that CCC had tremendous anti-obesity effects in the HFD-induced obese rats.

### CCC inhibited adipogenesis gene expression in the HFD-induced obese rats

Next, we examined the gene expression responsible for lipogenesis in the epididymal fat tissue. In agreement of with our *in vitro* cell culture studies, CCC decreased the phosphorylation of Akt, whereas wild type Akt was expressed at similar levels in all of the treated groups (Fig. 5D). The expression of PPARγ was decreased in the CCC-treated rats in a dose-dependent manner when compared to the HFD group (Fig. 5D).

## Discussion

Obesity is induced by increases in adipose tissue mass, which results from the multiplication of fat cells through adipogenesis and increased deposition of cytoplasmic triglycerides [29]. A reduction of adiposity is related to the inhibition of adipogenesis, along with a reduction in the number of adipocytes and the lipid content (cell size) of the adipocytes [30]. In the present study, we demonstrated that CCCE significantly inhibited adipogenesis and adipocyte differentiation in 3T3-L1 cells through the regulation of PPARγ activation and the Akt signaling pathway, leading to decreases in body weight and fat tissue mass in HFD-induced obese rats.

Lipid accumulation reflects the differentiation of preadipocytes into adipocytes, and this process is regulated by the increased expression of various transcription factors and adipogenesis-related genes [31]. The *transcription factors in adipocytes play critical roles* in mediating *adipogenesis and adipocyte differentiation*. The activation of C/EBPβ and C/EBPδ, which are key adipogenic transcription factors, was rapidly induced during an early stage of 3T3-L1 differentiation. C/EBPα and PPARγ are transcription factors expressed in the mid and late phases of differentiation and are known to activate adipocyte genes during the formation of mature adipocytes and to regulate insulin sensitivity. Therefore, in this study, we investigated whether CCC can affect adipocyte differentiation by measuring lipid accumulation and the expression of several genes associated with adipocyte differentiation and lipid metabolism in 3T3-L1 cells. CCC treatment remarkably attenuated the level of Oil-Red O staining in a dose-dependent manner, and microscopic inspection also revealed a significant decrease in the levels of accumulated intracellular triglycerides without affecting viability. This result suggested that CCC inhibited adipogenesis during adipocyte differentiation by causing an effective decrease in lipid formation and by reducing lipid accumulation in 3T3-L1 adipocytes. Moreover, CCC possesses the potential to down-regulate C/EBPβ, C/EBPδ and PPARγ mRNA expression in a concentration-dependent manner during the adipocyte differentiation of 3T3-L1 preadipocytes. Our data also showed that CCC markedly decreased the protein levels of C/EBPβ and PPARγ compared to fully differentiated adipocytes. Taken together, the lipid accumulation was directly proportional to the expression levels of PPARγ and C/EBPs in these cells treated with different concentrations of CCC. These results clearly implied that CCC had the ability to suppress adipocyte differentiation through the down-regulation of PPARγ and the C/EBPs. The expression of C/EBPα was not decreased by treatment with CCC, suggesting that the CCC-mediated anti-adipogenesis effects were caused by the inactivation of PPARγ, but not C/EBPα.

PPARγ is the master regulator of adipogenesis and controls the adipogenic process. Forced expression of PPARγ is sufficient to induce adipocyte differentiation in fibroblasts, and no factor has been discovered that promotes adipogenesis in the absence of PPARγ [12]. PPARγ activates the expression of the downstream adipocyte-specific genes aP2, LPL, and adiponectin [7], [32]. In this study, CCC significantly induced the down-regulation of PPARγ during 3T3-L1 differentiation, suggesting the inhibition of adipogenic and adipocyte-specific genes, such as aP2, LPL, leptin, and adiponectin. Therefore, we investigated the effect of CCC on the regulation of the PPARγ target genes, aP2, LPL, and adiponectin. The expression levels of LPL, adiponectin, and aP2 were dose-dependently suppressed by treatment with CCC. LPL catalyzes the hydrolysis reactions of triglycerides (TG), in which plasma TGs are metabolized into free fatty acids for TG synthesis by adipose cells [33]. Adiponectin is exclusively secreted from adipose tissue, which results in increased glucose uptake and fatty acid oxidation, and has been shown to improve insulin sensitivity [34]. The aP2 gene is a terminal differentiation marker of adipocytes, and it facilitates the cellular uptake of long-chain fatty acids in a pathway linking fatty acid metabolism and obesity [35]. Thus, these results suggested that the down-regulation of aP2, LPL, and adiponectin decreased fatty acid utilization and triglyceride synthesis in 3T3-L1 cells, and this, in turn, was able to inhibit adipocyte differentiation through the suppression of PPARγ activity. Moreover, CCC directly inhibited PPARγ transcriptional activity and preadipocyte differentiation in a concentration-dependent manner. Therefore, our results strongly suggested that CCC prevented adipogenesis through the inhibition of the signaling pathways involving PPARγ and through the reduced expression of adipogenesis- and lipid metabolism-associated genes in 3T3-L1 adipocytes.

The insulin signaling pathway plays an essential role in 3T3-L1 adipocyte differentiation [36]. The serine/threonine kinase Akt is particularly important in mediating adipocyte differentiation and the metabolic actions of insulin. GSK3β is a critical downstream signaling protein of the phosphoinositide 3-kinase (PI3K)/Akt pathway. Several studies of Akt signaling have implicated it in the regulation of PPARγ expression and adipocyte differentiation [12], [36], [37]. Moreover, Akt regulates adipogenesis via the phosphorylation and inactivation of substrates, such as GSK3β, which directly regulates PPARγ, C/EBPβ, C/EBPα and β-catenin [16], [38]. We predict that cross talk between Akt/GSK3β and PPARγ signaling might be a possible target for CCC that results in the disruption of adipocyte differentiation. In this study, these results showed that the insulin-MDI mixture increased the phosphorylation of Ser 473 on Akt and Ser 9 on GSK3β after MDI addition. However, the serine phosphorylation of Akt was decreased following CCC treatment in a dose-dependent manner, which subsequently attenuated the levels of phosphorylated GSK3β (Ser 9). These data indicated that inhibiting Akt phosphorylation reduced the phosphorylation of the downstream signaling components. Thus, our results strongly demonstrated that insulin-mediated Akt phosphorylation and activation was inhibited by treatment with CCC extract, which mainly affected the reduced accumulation of triglycerides by inhibiting the PI3K/Akt pathway during the differentiation of 3T3-L1 preadipocytes into adipocytes. Interestingly, GSK3β is a critically important protein kinase in adipocyte differentiation because it phosphorylates either C/EBPβ or C/EBPα. The inhibition of GSK3β phosphorylation (Ser 9) leads to C/EBPβ phosphorylation and inactivation [39], which is consistent with the negative regulation of C/EBPβ by GSK3β phosphorylation. Additionally, other studies have demonstrated that the phosphorylation of GSK3β (Ser 9) increases following insulin treatment, and its activity is repressed by insulin and lithium chloride (LC) [40]. The addition of LC to the differentiation medium of 3T3-L1 cells inhibited PPARγ expression and adipocyte differentiation [40]. Akt signaling also promotes adipocyte differentiation through an increase in PPARγ expression [41]. Conversely, inhibition of the Akt pathway in adipocyte differentiation has been shown to inhibit adipogenesis by decreasing PPARγ expression. The forced expression of PPARγ in Akt-deficient mouse embryonic fibroblasts rescued their severe adipogenesis defect [12], [37], which supports the essential role of PPARγ induction downstream of Akt. Therefore, our results suggested that the expression of the major transcription factor PPARγ as associated with the Akt pathway, and the inhibition of Akt phosphorylation and activation by CCC blocked the insulin-induced adipocyte differentiation of 3T3-L1 preadipocytes. Moreover, co-treatment with the PI3K/Akt inhibitor LY294002 and CCC resulted in a more significant inhibitory effect on triglyceride accumulation in 3T3-L1 cells when compared to LY2904002 treatment alone. In addition to its ability to block adipocyte differentiation of 3T3-L1 cells, we found that CCC inhibited Akt phosphorylation and significantly reduced insulin-stimulated glucose uptake, indicating that Akt was definitely involved in the inhibitory effect of CCC on glucose uptake in 3T3-L1 cells. Taken together, these results strongly indicated that CCC suppressed the adipogenic induction of lipid accumulation by inhibiting PPARγ activation through the suppression of the PI3K/Akt-signaling pathway during adipocyte differentiation process.

In the present study, we also used the HFD-induced obesity rat model to verify the anti-obesity effects of CCC extract *in vivo*. The body weights of the HFD-induced obese rats were monitored after daily oral administration of CCC at 60 or 200 mg/kg for 6 weeks. Interestingly, CCC caused a decrease in weight gain in the HFD rats after 6 weeks without affecting the food intake. Moreover, we observed that rats fed a HFD supplemented with CCC had significantly decreased levels of serum TG and TC compared with rats fed a HFD alone, indicating that CCC efficiently regulated TG and cholesterol metabolism in the HFD-induced obese rats. This raises the possibility of an anti-obesity mechanism by which CCC affects adipogenesis and energy expenditure. TC, which represents a combination of low-and high-density lipoprotein (LDL and HDL, respectively) cholesterol circulating in the blood, is one of the most commonly examined measurements in a lipid profile. HDL-cholesterol is considered good cholesterol, and high levels of HDL are a good indicator of a healthy heart because less cholesterol is available to attach to blood vessels; additionally, HDL affects the cholesterol efflux capacity. In general, a HFD has been associated with elevated serum LDL cholesterol and TGs levels [42]. We demonstrated that rats fed a HFD had significantly decreased serum HDL-cholesterol levels, while a HFD supplemented with CCC increased the serum HDL-cholesterol levels compared to the regular diet (RD) rats.

Similar to its effect on serum TG, CCC remarkably lowered the adipose tissue mass in rats fed a HFD supplemented with CCC compared to rats fed a HFD alone. The total fat volume of the epididymal and perirenal adipose tissues of representative HFD-induced obese rats increased by 1.9-fold compared with that of rats fed the regular diet (RD). However, the epididymal and perirenal fat volume of the rats treated with 60 or 200 mg/kg of CCC decreased by 15 or 25%, respectively, compared to that of the HFD obese rat. The CCC supplement induced significant losses in body weight, largely due to a reduction in epididymal and perirenal fat tissues. This result was consistent with a previous report, which demonstrated that the lipids in adipose tissue are largely derived from circulating TGs, especially during HFD feeding [43], and a reduction in serum TG levels also leads to decreased adipose tissue mass [44]. In epididymal adipocyte tissue, where the effects of CCC were observed most, treatment with CCC reduced the adipocyte size and lipid accumulation. Hypertrophy (large adipocyte) has been strongly correlated with diet, whereas hyperplasia is dependent on genetics. Recent evidence indicates that adipose tissue in mice exhibits dynamic remodeling when the animals are maintained on a HFD, with marked changes in adipocyte cell number and size occurring after high fat feeding [45]. In this study, a high fat diet caused the hypertrophy of the adipocytes, and treatment with CCC strongly reduced the hypertrophy in the HFD rats. In the liver of HFD-fed rat, we found that the HFD supplemented by CCC dramatically reduced hepatic lipid accumulation compared to HFD alone. Taken together, we suggested that the reduction of adipose tissue mass in rats fed a HFD supplemented with CCC caused the significant reductions in body weight gain, indicating that CCC administration influenced adipose tissue metabolism in obese rats. The differences in fat weight between the HDF and HFD+CCC rats might be due to the size of the adipocytes, which reflects the amount of lipid accumulation in the adipocytes. Importantly, CCC significantly down-regulated the expression of PPARγ and the phosphorylation of Akt in the HFD rats, which might be mechanisms that prevent the HFD-induced obese rats from gaining weight.

Numerous studies have indicated that various natural extracts inhibit body fat and lipid accumulation *in vitro* and *in vivo*. This study showed that CCC has an inhibitory effect on HFD-induced obesity. Although several studies have reported that CC extract exerts a variety of pharmacological activities, including hypolipidemic, antitumor, and antibacterial activities, no study has examined the ability of CCC to prevent obesity in 3T3-L1 cells and HFD obese rats. In this study, we found that CCC contained high total phenolic and flavonoid contents and was effective at scavenging for DPPH, superoxide anion, and hydroxyl radicals, which correlated with its antioxidant potential. Considering a potential use of antioxidant substances as anti-obesity agents, the antioxidant potential of CCC can prevent adipocyte differentiation in 3T3-L1 cells and HFD obese fat tissues.

In the present study, we investigated the anti-obesity effects of CCC on adipocyte differentiation and associated mechanisms in 3T3-L1 cells and confirmed our finding in an obese rat model fed a HFD. CCC greatly reduced the expression of C/EBPβ and C/EBPδ and subsequently down-regulated the activation of the key transcriptional regulator PPARγ in 3T3-L1 adipocytes. The levels of Akt and GSK3β phosphorylation were significantly attenuated by CCC treatment, which blocked adipogenesis and adipocyte differentiation in 3T3-L1 cells. Moreover, the administration of CCC effectively suppressed HFD-induced body weight gain and decreased the body fat and lipid content in the tissues of the rats. These studies indicated that the anti-obesity effects of CCC were accompanied by a significant decrease in phospho-Akt and PPARγ expression in HFD tissues, thus suggesting that CCC has the ability to prevent HFD-induced obesity, possibly through the activation of Akt and PPARγ signaling. These results suggested that the anti-obesity effect of CCC results from a decrease in adipogenesis and that CCC is a good candidate for an anti-obesity agent.
